# Auxin alone provokes retention of *ASH1* mRNA in *Saccharomyces cerevisiae* mother cells

**DOI:** 10.17912/micropub.biology.000752

**Published:** 2023-02-22

**Authors:** Ginola Domeni Zali, María Moriel-Carretero

**Affiliations:** 1 Centre de Recherche en Biologie cellulaire de Montpellier (CRBM), Université de Montpellier, Centre National de la Recherche Scientifique, 34293 Montpellier CEDEX 05, France

## Abstract

The auxin-inducible degradation (AID) system can elicit conditional and reversible protein degradation as a tool to assess the role of essential proteins. Indeed, AID enables functional studies without the possibility of adaptation, which can occur with permanent gene deletions. The AID system relies on the addition of auxin molecules, such as indole-3-acetic acid (IAA), as a means to launch the degradation of the protein of interest. In this context, it is extremely important to control for the effect of auxin addition alone. To study the role of essential proteins in the process of selective mRNA delivery to daughter cells in *Saccharomyces cerevisiae* , we first controlled for the effect of adding IAA to cells that cannot perform AID-mediated degradation. We found that auxin alone restricted *ASH1* delivery to daughter cells, as *ASH1* mRNA started being retained in the mother cell as soon as thirty minutes after IAA addition. Thus, our data warn about the danger of not systematically including auxin-treated cells incapable of degradation in every AID-related experiment. Furthermore, given previous data reporting the ability of auxin to inhibit the master growth regulator TORC1 in *S. cerevisiae* , our data suggest that TORC1 could control the selective delivery of mRNAs to daughter cells.

**Figure 1. Effect of IAA on the segregation of the ASH1 mRNA to daughter cells:**
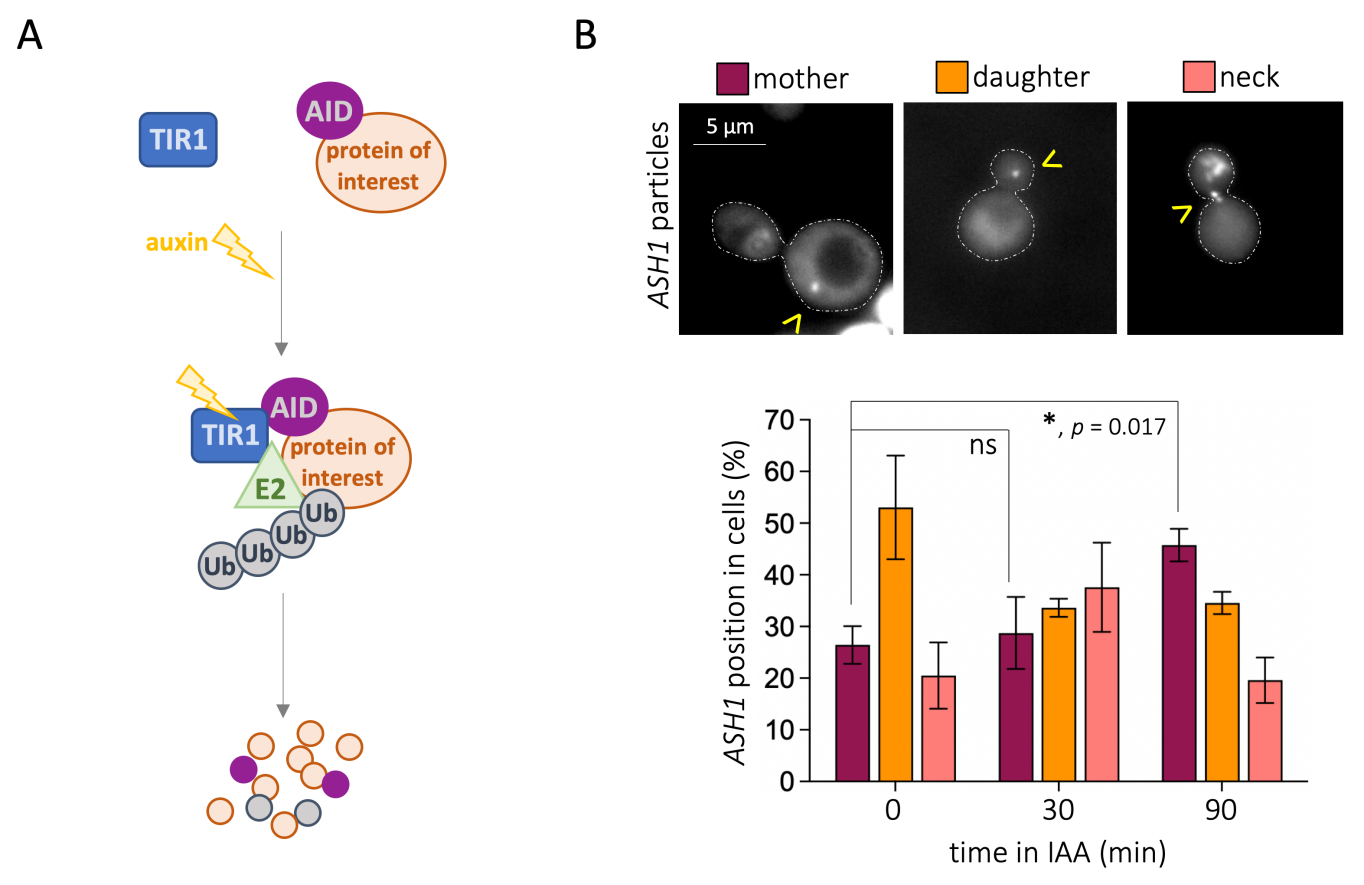
**A)** Schematic illustration of the AID system. Briefly, a protein of interest is tagged with the AID motif in a cell that is simultaneously expressing the E3 ubiquitin ligase subunit TIR1. Auxin addition then causes TIR1 to interact with the AID motif, targeting the AID-tagged protein for ubiquitination, as this will bring ubiquitin (Ub)-activating E2 enzymes into proximity. Ubiquitination of the protein of interest will target it for proteasomal degradation. **B)** **Top:** Representative images of yeast cells displaying *ASH1* mRNA foci in the mother, in the daughter, and in the neck. The *ASH1* mRNA particles are observed as foci, indicated by yellow arrowheads. **Bottom:** Quantitative analysis of the distribution of mRNA foci located in the mother, in the neck, or in the daughter in the absence of IAA or after the indicated times after its addition. At least 50 foci, and never less than 400 cells, were counted per condition, timepoint, and experiment. The bar height represents the mean value out of 3 independent experiments and error bars indicate the standard error of that mean. The *p* -value (ns = non-significant) was derived by unpaired one-way ANOVA between the concerned means.

## Description

Researchers have various tools to study protein function. One such tool is the auxin-inducible degron (AID) system, which allows conditional, transient, and reversible degradation of suitably tagged proteins (Nishimura *et al.* 2009). Auxins are hormones, derived from the amino acid tryptophan, that control gene expression during many aspects of plant growth and development (Leyser 2018). Auxins can be natural, such as indole-3-acetic acid (IAA), or synthetic, such as 1-naphthaleneacetic acid (NAA). AID works through the evolutionarily conserved ubiquitin ligase-related degradation pathway, allowing its use as a genetic tool in yeast and mammalian cells (Nishimura *et al.* 2009). Thus, an auxin-inducible destabilizing (AID) domain, or «degron» can be fused either to the amine or carboxy terminus of a target protein to elicit its destruction. To be effective, the AID system requires the expression of the F-box transport inhibitor response 1 (TIR1) protein. The presence of auxin will mediate the interaction between the AID-tagged protein and TIR1, thus inducing polyubiquitination and rapid proteasomal degradation of the degron-fused protein (Nishimura *et al.* 2009) (Figure 1A). Thus, to unravel the discrete phenotypes associated with the lack of a protein of interest, cells are exposed to auxin and, with variable kinetics ranging from minutes to several hours, its degradation will be achieved. Pertinent controls need to be included in the experimental set-up: it is important to use auxin to treat both cells that have the genetic components necessary to trigger the degradation response and those that do not. This will ensure no phenotype can be accounted for by auxin alone.

In eukaryotes, cell polarity is established via the asymmetric distribution of messenger RNAs (mRNAs). In the model organism *Saccharomyces cerevisiae* , this polarization manifests as the budding of daughter cells, and more than thirty mRNAs have been identified as selectively targeted (Shepard *et al.* 2003; Aronov *et al.* 2007). Different mRNAs are segregated at different cell cycle stages. For example, *WSC2* , *IST2,* or *EAR1* mRNAs are delivered when the bud starts to emerge during S / G _2_ phases, whereas *ASH1* mRNA is distributed late during mitosis (Shepard *et al.* 2003; Fundakowski *et al.* 2012). *ASH1* subcellular localization and dynamics have been studied by modifying its sequence to include a MS2 binding site and co-expressing a fluorophore-fused MS2 protein (Bertrand *et al.* 1998). The myosin Myo4p ensures the trafficking of selectively targeted mRNAs along actin filaments. Myo4p is recruited as part of the “locasome” complex that includes the RNA-binding protein, She2p and the adaptor protein She3p (Münchow *et al.* 1999; Bohl *et al.* 2000; Long *et al.* 2000; Takizawa and Vale 2000), although She2p-independent delivery events have been reported (Samardak and Moriel-Carretero 2021).

We were interested in exploring the putative role of essential factors in this selective delivery of mRNAs to daughter cells, therefore we turned our attention to the AID system. First, we performed controls in which cells lacking all the needed AID system components, thus unable to degrade any protein, were subjected to auxin. These cells still harbored the MS2 system allowing us to monitor *ASH1* subcellular distribution. We counted the *ASH1* foci located in the mother cell, in the daughter cell, and those located in the neck between them (Figure 1B, top panel). At basepoint, the percentage of *ASH1* foci was higher in daughter cells (55 %) than in the mother (25%) and the neck (20%). We then added 1 mM IAA and assessed foci distribution at 30 and 90 min. At 30 min there was already a progressive, though not significant, stalling of *ASH1* particles at the neck at the expense of the daughter cells (Figure 1B, bottom, 40% in the neck). By 90 min the pattern was completely inverted, with almost 50% of mRNA foci found in the mother (Figure 1B, bottom). These data suggest that the transport of these mRNAs to the daughter cells is significantly less efficient in the presence of IAA (Figure 1B, bottom, asterisk).

In the first instance then, our results highlight the importance of using a control group to monitor any potential side effect of auxin addition, as initially preconized by (Nishimura *et al.* 2009). Although examples exist where this precaution was fortunately implemented (Li *et al.* 2022), we urge the community never to neglect it, even if the model under study does not possess a canonical auxin-responsive system. Indeed, auxin alone can exert important physiological changes. For example, Nicastro et al. demonstrated that *Saccharomyces cerevisiae* is sensitive to extracellular IAA, which limits its growth by inhibiting the target of rapamycin complex 1 (TORC1), both *in vivo* and *in vitro* (Nicastro *et al.* 2021), and IAA was shown to activate filamentation thus invasive growth in this same fungus (Prusty *et al.* 2004). Furthermore, little research has been conducted on the effects of auxin alone in humans, thus the absence of effects should not be assumed. For example, IAA, at a dose identical to the one used here, has been reported to cause marked ultrastructural changes and death of neutrophils and lymphocytes (de Melo *et al.* 2004). Thus, the omission of auxin-only controls may lead to misinterpretation and wrong conclusions.

A second added benefit of our data presented here is that it provides a new insight into the field of selectively delivered mRNAs. The finding that auxin alters the segregation of *ASH1* mRNA to the daughter cell may have further significance: although this effect could be specific to *ASH1* mRNA, it is possible that auxin might also perturb the delivery of other mRNAs. In that case, the system would be ineffectual for functional studies regarding the selective delivery of mRNAs, at least for those targeted to the daughter cell, and this needs further testing. Last, knowing that auxin is a known inhibitor of TORC1 (Nicastro *et al.* 2021), our observation points to TOR as a potential master regulator of selective mRNA delivery to the daughter cell. Given that TOR inhibition is a means to halt cell growth and proliferation (Loewith and Hall 2011), simultaneous lack of daughter cell specification suggests a fine coordination to restrain cell division. To our knowledge, this possible role of TOR has never been raised before, although we are aware that IAA-mediated TOR inhibition may not be the only explanation for the effect of auxin on *ASH1* localization.

## Methods

*Saccharomyces cerevisiae* cells were grown at 25°C in selective YNB liquid medium supplemented with 2% glucose without histidine to ensure plasmid maintenance. All experiments were performed with exponentially growing cells. For microscopy analyses, 1 mL of the culture of interest was centrifuged; then, the supernatant was discarded, and the pellet was resuspended in the remaining 50 μL. Next, 3 μL of this cell suspension was directly mounted on a coverslip for immediate imaging of fluorescent signals using the requisite wavelength. Imaging was achieved using a ZEISS Axio Imager 2 microscope and Metamorph software. Subsequent image visualization and analysis were performed with Image J v2.0.0-rc-69/1.52i. The determination of the distribution of *ASH1* foci within cells was performed by visual inspection. GraphPad Prism was used to plot and statistically analyze the results.

## Reagents

The used strain (TC-230, *RFA1* -AID*-myc9-G418 ^R^ , *ASH1* -12MS2L) possesses a modification of the *ASH1 locus* allowing the expression of an *ASH1* mRNA bearing 12 loops in its 3’ untranslated region that can be bound by the MS2 protein. This strain was transformed with plasmid RJP1486 (pCP-MS2-3xGFP- *HIS3* ). Both the plasmid and the original YRJ3525 strain with the *ASH1* modification were kind gifts from Pr. Ralf-Peter Jansen, Tübingen University. IAA reference is I2886 from Sigma-Aldrich.
